# Colorectal cancer stem cell and chemoresistant colorectal cancer cell phenotypes and increased sensitivity to Notch pathway inhibitor

**DOI:** 10.3892/mmr.2015.3694

**Published:** 2015-04-28

**Authors:** RUI HUANG, GUIYU WANG, YANNI SONG, QINGCHAO TANG, QI YOU, ZHENG LIU, YINGGANG CHEN, QIAN ZHANG, JIAYING LI, SHAN MUHAMMAND, XISHAN WANG

**Affiliations:** 1 Department of Colorectal Surgery, The Second Affiliated Hospital of Harbin Medical University, Harbin, Heilongjiang 150086, P.R. China; 2 Department of Breast Surgery, The Third Affiliated Hospital of Harbin Medical University, Harbin, Heilongjiang 150081, P.R. China; 3 Colorectal Cancer Institute of Harbin Medical University, Harbin, Heilongjiang 150086, P.R. China

**Keywords:** colorectal cancer, cancer stem cells, chemoresistant cells, Notch pathway, CD133/CD44

## Abstract

Colorectal cancer stem cells (Co-CSCs) are a small subpopulation of tumor cells which have been proposed to be tumor-initiating cells in colorectal cancer (CRC) and to be implicated in resistance to standard chemotherapy. Chemoresistance is a common problem in the clinic. However, the interrelation between Co-CSCs and chemoresistant cells has yet to be elucidated. The present study investigated the Co-CSC phenotype in colonospheres and chemoresistant CRC cell lines and aimed to identify targets for therapy. Colonospheres and chemoresistant CRC cells were found to be enriched with the CSC markers CD133 and CD44, and exhibited similar phenotypes. Furthermore, it was found that Notch signaling may simultaneously regulate Co-CSCs and chemoresistant cells and may represent a novel strategy for targeting this pathway in CRC.

## Introduction

Colorectal cancer (CRC) is a major cause of cancer mortality and morbidity worldwide. It is the third most commonly diagnosed cancer in males and the second in females, with >1.2 million new cases and 608,700 mortalities estimated to have occurred in 2008 (1). At present, the identification of novel strategies for therapy is urgently required.

The colorectal cancer stem cell (Co-CSC) hypothesis provides a novel understanding of tumorigenesis. Increasing evidence shows that Co-CSC subpopulations are capable of self-renewal, driving tumor growth and differentiating to form all of the lineages observed within a tumor (2–5). Therefore, Co-CSCs may represent a novel target for the treatment of tumors (6). Current strategies for cancer treatment require modification to ensure that the Co-CSCs which are driving tumor growth are specifically targeted. However, there is increasing evidence that Co-CSCs are more resistant to current chemotherapy (7) than other subpopulations of cells within the tumor. This may be one of the reasons why the effectiveness of standard chemotherapy is limited. Standard chemotherapeutics are incapable of eradicating Co-CSCs, potentially due to drug efflux, autocrine survival signaling and alterations in DNA damage repair mechanisms in Co-CSCs (8,9). However, the specific mechanism underlying Co-CSC chemoresistance has yet to be elucidated.

5-fluorouracil (5FU) and oxaliplatin are the predominant chemotherapeutic agents used for treating advanced CRC. 5FU and oxaliplatin have different mechanisms of action. 5FU inhibits the activity of the thymidylate synthase enzyme during DNA replication (10), while oxaliplatin causes prolonged G_2_-phase arrest and inhibits tumor cell growth through covalent DNA binding (11). Although the mechanisms of tumor cell resistance to 5FU and oxaliplatin have been extensively investigated, the specific mechanism remain to be fully elucidated. Previous studies have reported that these chemoresistant cells overexpress markers of CSCs (12). This shows that chemoresistant tumor cells represent a subpopulation of cells from the original tumor which are molecularly and phenotypically distinct. Therefore, the association between Co-CSCs and chemoresistant cells is of interest. Co-CSCs exhibit characteristics of cells which are resistant to standard chemotherapeutics and chemoresistant cells also express markers of Co-CSCs. Therefore, Co-CSCs and chemoresistant cells may be interrelated. Thus, the identification of a common target of Co-CSCs and chemoresistant cells may be of significance for clinical treatment.

A number of important signaling pathways, including Wnt, Notch and Hedgehog, have been found to have a role in regulating the self-renewal of CSCs in the hematopoietic system, skin, nervous system and breast (13–15). However, the mechanism by which pathways, including the Notch pathway, regulate Co-CSCs and chemoresistant cells, as well as the role that Notch has in the interrelation between these two types of cells, has yet to be elucidated.

Based on the clinical significance of chemoresistance and the ineffectiveness of chemotherapy in eliminating Co-CSCs, the present study aimed to investigate the interrelation between Co-CSCs and chemoresistant cells. Molecular and phenotypic alterations were investigated in Co-CSCs and chemoresistant cells *in vitro*, as well as using a mouse model system *in vivo*. Notch pathway activation was also detected in the Co-CSCs and chemoresistant cell lines and was targeted using xenograft models. The present study has two clinical implications associated with the interrelation between Co-CSCs and chemoresistant cells. The first is that it translates the theory of CSCs into clinical practice and shows that similar mechanisms may act in Co-CSCs and chemoresistant cells. The second is that it shows that Notch signaling simultaneously regulates Co-CSCs and chemoresistant cells and identifies a novel mechanism of targeting the Notch signaling pathway in CRC. Of note, the altered Notch activity observed in the present study may partially explain the chemoresistance in Co-CSCs and chemoresistant cells.

## Materials and methods

### Cell lines and culture

The HCT116 human CRC cell line was obtained from the Colorectal Cancer Institute of Harbin Medical University (Harbin, China). Colonospheres were cultured as described previously (16). In brief, using a limited dilution method, 1–3 cells were seeded on a 96-well ultralow-attachment plate (Corning Life Sciences, Corning, NY, USA) with Dulbecco’s modified Eagle’s medium (DMEM)/F12 medium containing B27 supplement (Invitrogen Life Technologies, Carlsbad, CA, USA), 20 ng/ml basic fibroblast growth factor and 20 ng/ml epidermal growth factor, which served as the stem cell medium (SCM) for the experiments. Under these culture conditions, Co-CSCs, but not differentiated cancer cells, are able to survive and proliferate (17–19). The number of surviving cells in each well of the 96-well plate was then observed and one cell well was selected and marked. After seven days, the cells were observed and the death cell well was removed and SCM was added to the survival cell well. After 10–14 days, the well in which colonospheres grew was marked and colonospheres were supplemented with SCM every three days until the colonospheres were able to be passaged.

An oxaliplatin-resistant cell line (HCT116/OxR) and 5FU-resistant cell line (HCT116/5FU-R) were developed as previously described (20,21). The resistant cells and parental cells were grown in DMEM containing 10% fetal bovine serum (FBS; Sigma-Aldrich, St. Louis, MO, USA) and 1% antibiotic solution (Mediatech Inc., Herndon, VA, USA) at 37°C in a humidified incubator containing 5% CO_2_.

### Drugs and antibodies

Oxaliplatin and 5FU were purchased from the Colorectal Cancer Institute of Harbin Medical University. The γ-secretase inhibitor *N*-[*N*-(3,5-difluoroph enacetyl)-l-alanyl]-*S*-phenylglycine *t*-butyl ester (DAPT) was purchased from Sigma-Aldrich and was used to inhibit Notch signaling *in vitro* and *in vivo*. The antibodies used for flow cytometry, immunohistochemistry (IHC), immunofluorescence and western blot analysis were as follows: Rabbit anti-cluster of differentiation (CD) 133, rabbit anti-Notch1 (Cell Signaling Technology, Inc., Beverly, MA, USA), rabbit anti-β-actin (Sigma-Aldrich), mouse anti-CD44 (Abcam PLC, Cambridge, UK), allophycocyanin (APC)-conjugated anti-CD133, APC-conjugated mouse-immunoglobulin G (IgG) 1 (Miltenyi Biotec, Bergisch Gladbach, Germany), phycoerythrin (PE)-conjugated anti-CD44, PE-conjugated mouse-IgG2b (Becton, Dickinson and Company, Franklin Lakes, NJ, USA), rabbit anti-hairy and enhancer of split-1 (HES-1; Santa Cruz Biotechnology, Inc., Santa Cruz, CA, USA) and mouse anti-Ki67 (Dako, Ely, UK).

### Proliferation and chemosensitivity

Cell proliferation and drug sensitivity were assessed using a Cell Counting Kit-8 (CCK-8) assay as described previously (22). In brief, the cell lines were seeded at a density of 5,000 cells/well in 96-well plates in 100 *µ*l media with or without 5FU, oxaliplatin or DAPT. At each time-point (0, 24, 48 and 72 h), 10 *µ*l CCK-8 solution was added to each well and incubated for a further 2 h. The absorbance was read at 450 nm using a standard microplate reader.

### Colonosphere assay

Each cell line was trypsinized and quantified by plating a single cell in each well of a low-attachment 96-well plate and counted under a microscope, assessing the rate of colonospheres. The colonospheres were cultured using the aformentioned process.

### Clonogenic assay

Clonogenic assays were performed to determine the proliferative capacity of the colonospheres and the chemoresistant cells. A total of 500 cells/well were seeded on a six-well plate and incubated for 14 days at 37°C in 5% CO_2_. Following incubation, the colonies were formalin-fixed and stained with hematoxylin. Colonies which were >50 *µ*m were counted under a light microscope (CKX31; Olympus, Tokyo, Japan) and compared with the parental cells.

### Western blot analysis

Cell lysates were subjected to SDS-PAGE and blotted onto Immobilon^®^-P polyvinylidene difluoride membranes (Millipore Corporation, Billerica, MA, USA). Specific proteins were detected using an enhanced chemiluminescence system (GE Healthcare, Little Chalfont, UK). Membranes were probed with the aforementioned antibodies.

### Flow cytometry

Single cells were prepared for the analysis of cell surface markers by digesting with pancreatin. Cells were then detached from the plates through incubation with enzyme-free cell dissociation buffer (Invitrogen Life Technologies). Cells were then washed with 10 mmol/l cold phosphate-buffered saline (PBS) and resuspended in 1X binding buffer (BD Biosciences, San Jose, CA, USA) at a concentration of 1×10^6^ cells/ml. Cells were subjected to direct immunofluorescence staining using APC-conjugated anti-CD133 and PE-conjugated anti-CD44 antibodies followed by flow cytometric analysis. Samples were analyzed using a NucleoCounter^®^ NC-3000 analyzer (ChemoMetec, Lillerød, Denmark). The experiments were repeated at least three times.

### In vivo assay and Notch pathway inhibition

Male nude mice, aged four weeks, were purchased from the Shanghai Laboratory Animal Center (Shanghai, China). All animal experiments were approved by the Institutional Animal Care and Use Committee of Harbin Medical University. Equal numbers of cells (1×10^6^) from colonospheres and chemoresistant cell lines were suspended in 100 *µ*l PBS and injected subcutaneously into the flank of each mouse (six mice per group). When the tumors reached ~100 mm^3^, mice were subjected to intraperitoneal injection with 200 *µ*g DAPT twice per week. Tumor growth was observed and recorded over 10 weeks. When the tumors in the control group exceeded 1.5 cm in diameter, the animals were euthanized and the tumors were weighed and measured. Tumor volume was calculated using the following formula: (length)/2×(width)^2^. Tumors were then paraffin-embedded, sectioned and stained with hematoxylin and eosin (H&E) for histological analysis. IHC and apoptotic analysis were also performed. Data are shown from representative experiments.

### Immunohistochemistry and immunofluorescent analysis

IHC was performed on 4-*µ*m sections which were prepared using the paraffin-embedded tissues. Following standard procedures, tissues were deparaffinized using xylene, hydrated in graded alcohol and pretreated for antigen retrieval in Tris/ethylene diaminetetracetic acid buffer for 5 min in a 100°C steamer. Slides were then H&E stained in order to assess morphology or incubated with anti-Ki67 antibodies to visualize the proliferative nuclei. All sections were developed using 3,3′-diaminobenzidine and counterstained with hematoxylin (Invitrogen Life Technologies). Apoptotic cells within the tissue were detected using the terminal deoxynucleotidyltransferase-mediated dUTP nick-end labeling (TUNEL) assay. An Apoptag *in situ* apoptosis detection kit (Roche Diagnostics, Mannheim, Germany) was used for TUNEL staining according to the manufacturer’s instructions for paraffin-embedded tissues. Immunohistochemical staining and fluorescence were analyzed using a Zeiss Axioskop microscope (Carl Zeiss AG, Oberkochen, Germany) and apoptosis was expressed as the percentage of TUNEL positive cells.

### Statistical analysis

All data are presented as the mean ± standard error of three independent experiments, each performed in triplicate. Data were analyzed using the Student’s t-test. Analysis of variance was performed for multiple comparisons. P<0.05 was considered to indicate a statistically significant difference. SPSS 17.0 statistical software (SPSS, Inc., Chicago, IL, USA) was used for the analyses.

## Results

### Expression of CSC markers in the colonospheres and chemoresistant cells

**Figure 1:**
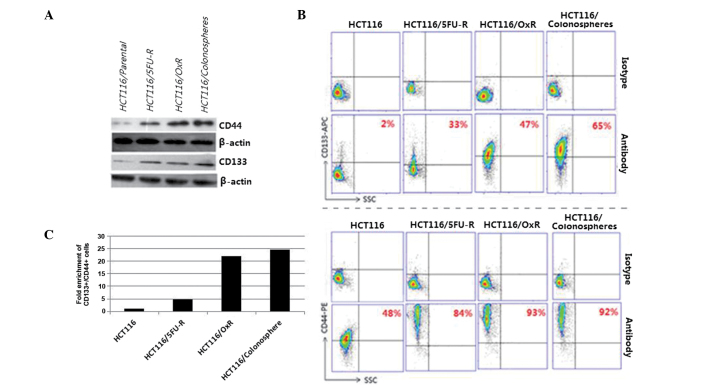
Colonospheres and chemoresistant cell lines are enriched with Co-CSC markers. (A) Western blot analysis revealed that expression of the Co-CSC markers CD133 and CD44 was higher in the colonospheres and HCT116/5FU-R and HCT116/OxR chemoresistant cells compared with the parental HCT116 human CRC cells. β-actin was used as a loading control. (B) Flow cytometric analysis revealed that the colonospheres and chemoresistant cell lines were enriched with cells expressing CD133 and CD44 compared with the parental cell line. A total of 33% of the HCT116/5FU-R cells, 47% of the HCT116/OxR cells and 65% of the HCT116/colonosphere cells expressed CD133 compared with 2% of the parental HCT116 cells. Similarly, 84% of the HCT116/5FU-R cells, 93% of the chemoresistant cells and 92% of the HCT116/colonosphere cells expressed CD44 compared with 48% of the parental cells. Cytometric analysis plots using isotype control antibodies were used as staining controls. (C) CD44 and CD133 labelling and flow cytometric analysis revealed a 4.8-, 22- and 24.7-fold enrichment of double-positive cells in the HCT116/5FU-R, HCT116/OxR and colonosphere cells compared with the parental HCT116 cell line. SCC, side scatter; Co-CSC, colorectal cancer stem cell; CD, cluster of differentiation; 5-FU, 5-fluorouracil; R, resistant; Ox, oxaliplatin.

CRC has been proposed to arise specifically in stem cell populations at the base of colonic crypts. Markers used for the identification of Co-CSCs include CD44, CD133, CD24, CD29, leucine-rich repeat-containing G-protein coupled receptor 5 and doublecortin-like kinase 1 (23). Among these markers, CD44 and CD133 have been widely used for the identification of CSCs in CRC. The CSC population has been reported to be capable of self-renewal and generating tumors resembling the primary tumor. Moreover, CSCs have been found to be capable of growth in serum-free medium and the formation colonospheres. In the present study, the expression profiles of HCT116 human CRC colonospheres and cells resistant to 5FU or oxaliplatin (HCT116/5FU-R or HCT116/OxR, respectively) were assessed using western blot analysis and flow cytometry. Compared with the parental HCT116 cells, CD133 and CD44 expression were observed to be significantly higher in the colonospheres, HCT116/5FU-R and HCT116/OxR cells (Fig. 1A). The number of cells expressing CD133 and CD44 was also found to be significantly higher in the colonospheres and chemoresistant cells compared with the parental cells (Fig. 1B), with only 2% of the parental cells expressing CD133 and 48% expressing CD44, while between 33 and 65% of the three cell types expressed CD133, and between 84 and 93% of the three cell types expressed CD44. Following CD133 and CD44 labeling, flow cytometric analysis revealed a 4.8-fold enrichment of CD133^+^/CD44^+^ cells in the HCT116/5FU-R cell line, a 22-fold enrichment of CD133^+^/CD44^+^ cells in the oxaliplatin-resistant cell line and a 24.7-fold enrichment of CD133^+^/CD44^+^ cells in the colonospheres compared with the parental HCT116 cells (Fig. 1C).

### Cell phenotype in the colonospheres and chemoresistant cells

**Figure 2:**
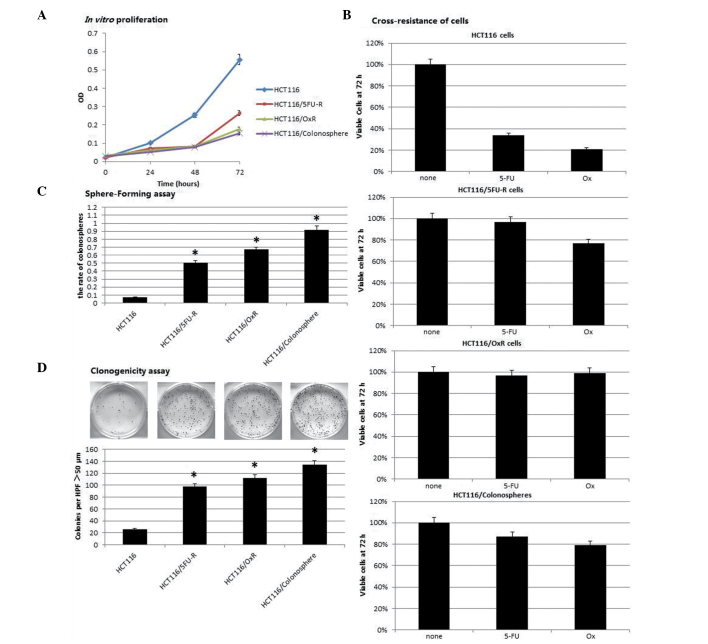
Colonospheres and chemoresistant cell exhibit a cancer stem cell phenotype. (A) Colonospheres and chemoresistant cells proliferated at a significantly slower rate than parental cells, detected using the Cell Counting Kit-8 assay. (B) Parental cells were sensitive to 5FU and Ox following exposure for 72 h, with only 34 and 21% of cells remaining, respectively, compared with the untreated cells. By contrast, HCT116/5FU-R cells were resistant to 5FU; however, they also exhibited increased resistance to Ox compared with the parental cells. Similarly, HCT116/Ox-R cells were resistant to Ox, as well as 5FU. Colonosphere cells were also resistant to Ox and 5-FU. (C) Cells were plated in an ultralow-attachment 96-well plate in the absence of serum and after 14 days, the rate of viable sphere-forming cells was assessed. The colonosphere formation rate was significantly increased in the colonospheres and chemoresistant cells compared with the parental cells. (D) Clonogenic assay revealed that the number of colonies larger than 50 *µ*m in diameter which were formed under standard growth conditions was significantly higher in the colonospheres and chemoresistant compared with the parental HCT116 cells. Data are presented as the mean ± standard error. ^*^P<0.05 vs. HCT116 cells. OD, optical density, 5-FU, 5-fluorouracil; Ox, oxaliplatin; R, resistant.

*In vitro* proliferation was assessed through plating an equal number of cells from each cell line and using a CCK-8 assay as an index of cell number. The proliferation rates of the colonospheres, 5FU- and oxaliplatin-resistant cells were found to be significantly lower than those of the parental cells (52–72%; P<0.05; Fig. 2A). The CCK-8 assay was also used to analyze cell sensitivity to chemotherapeutic agents. Colonospheres, 5FU- and oxaliplatin-resistant cells were exposed to clinically relevant doses of 5FU and oxaliplatin. The number of cells remaining after 72 h was then assessed. Parental cells were found to be sensitive to oxaliplatin and 5FU, with only 34 and 21% of the cells remaining viable following exposure to oxaliplatin and 5FU, respectively (Fig. 2B). 5FU-resistant cells were observed to be resistant to 5FU; however, these cells were also resistant to oxaliplatin, with 77% of the cells remaining after 72 h of exposure. Similarly, oxaliplatin-resistant cells were found to be resistant to oxaliplatin, but also exhibited cross-resistance to 5FU. Colonospheres were resistant to oxaliplatin and 5FU, with 79–87% of the cells remaining viable after 72 h of exposure.

CSCs have the capacity to form colonies, also known as spheres, in the absence of serum and without attachment to culture plates. In the present study, the capacity of colonospheres and chemoresistant cell lines to grow colonospheres under serum-free conditions was analyzed. Cell lines were trypsinized and quantified by plating a single cell in each well of a low-attachment 96-well plate and assessing the capacity of the cells to form colonospheres. In the colonosphere cells, the rate of secondary sphere generation was higher than that in the HCT116/5FU-R and HCT116/OxR cells. However, compared with the parental cells, an increased number of colonospheres was found in the three cell types (P<0.05; Fig. 2C). The clonogenic assay revealed that the three types of cells exhibited an increased colonosphere formation capacity after 14 days of culture (Fig. 2D).

### Notch pathway in colonospheres and chemoresistant cells

**Figure 3:**
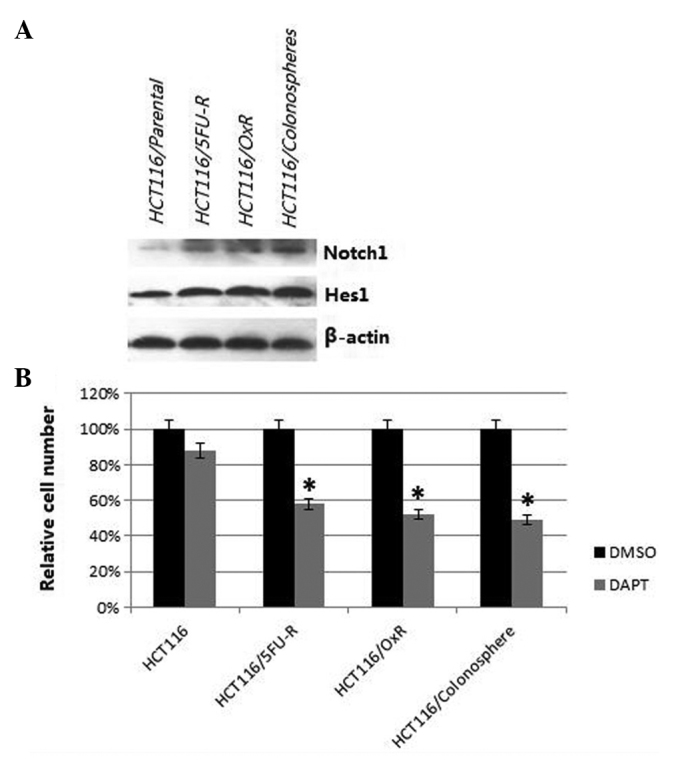
Effect of Notch pathway inhibition on colonospheres and chemoresistant cells. (A) Analysis of whole-cell lysates from parental HCT116 cells, chemoresistant HCT116/5FU-R and HCT116/Ox-R cells and colonosphere cells revealed an increase in Notch1 and Hes1 in the chemoresistant and colonosphere cells compared with the parental cells. (B) Cells were treated with DMSO (control) or a Notch pathway inhibitor DAPT and the cell number was assessed. Upon DAPT treatment, the cell number was significantly decreased in the chemoresistant and colonosphere cells (42–51%) compared with the HCT116 cells (12%). ^*^P<0.05 vs. HCT116 cells. 5-FU, 5-fluorouracil; R, resistant; Ox, oxaliplatin; DAPT, *N*-[*N*-(3,5-difluorophenacetyl)-l-alanyl]-*S*-phen ylglycine *t*-butyl ester; DMSO, dimethyl sulfoxide; Hes, hairy and enhancer of split.

Western blot analysis was used to assess constitutive signaling in the parental, colonosphere and chemoresistant cell lines, with a focus on targets for which agents that inhibited target function were readily available. Several signaling pathways were investigated, but the most marked alterations were observed in the Notch signaling pathway, thus the present study focused on Notch signaling. Notch1 levels were found to be higher in the colonospheres and chemoresistant cell lines compared with the parental cells (Fig. 3A). Furthermore, the levels of hairy and enhancer of split 1 (Hes1) were also observed to be increased in the colonospheres and chemoresistant cell lines compared with the parental cells.

DAPT, a γ-secretase inhibitor, was used to determine the dependence of the cells on Notch signaling for survival. The CCK-8 assay revealed that DAPT treatment caused a minor decrease (12%) in cell number in the parental cells, but a significantly greater reduction in cell number in the colonospheres and chemoresistant cells compared with the parental cells (42% for HCT116/5FU-R, 48% for HCT116/OxR and 51% for HCT116/colonospheres; all P<0.05; Fig. 3B).

### Effect of Notch pathway inhibition on in vivo tumor growth

**Figure 4:**
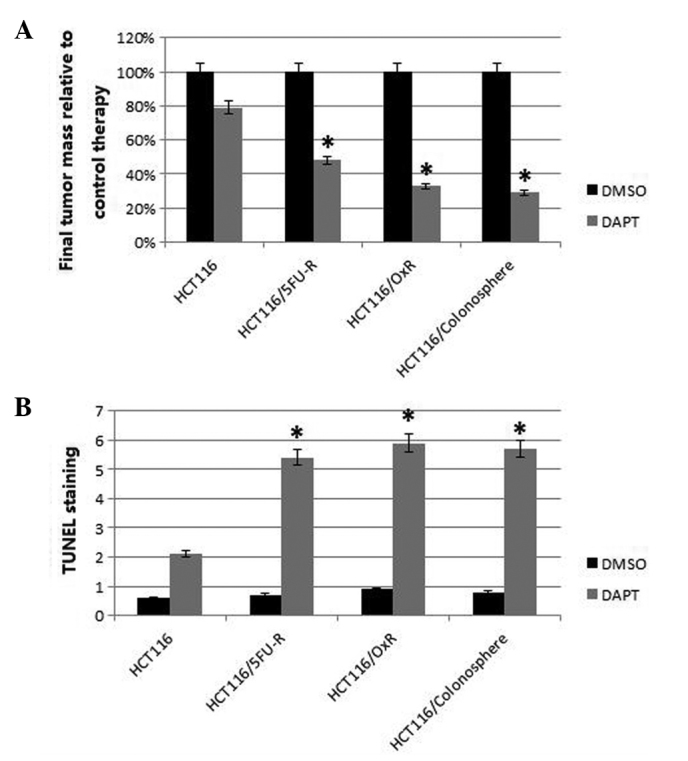
Effect of Notch pathway inhibition on *in vivo* tumor growth, proliferation and apoptosis. Mice were subcutaneously injected with 1×10^6^ HCT116, HCT116/5FU-R, HCT116/OxR or HCT116/colonosphere cells and treated with DMSO (control) or DAPT twice weekly. Final tumor masses were measured and compared between mice bearing tumors from each cell line. (A) In the DAPT-treated mice, the HCT116/5FU-R-, HCT116/OxR- and HCT116/colonosphere-derived tumors showed significantly greater growth inhibition than the HCT116-derived tumors. (B) In the DAPT-treated mice, TUNEL staining revealed significantly greater apoptosis in the HCT116/5FU-R-, HCT116/OxR- and HCT116/colonospheres-derived tumors than in tumors derived from the HCT116 cells. Data are presented as the mean ± standard error. ^*^P<0.05 vs. HCT116 cells. 5-FU, 5-fluorouracil; R, resistant; Ox, oxaliplatin; DAPT, *N*-[*N*-(3,5-difluorophenacetyl)-l-alanyl]-*S*-phenylglycine *t*-butyl ester; DMSO, dimethyl sulfoxide; TUNEL, terminal deoxynucleotidyltransferase-mediated dUTP nick-end labeling.

Colonospheres and chemoresistant cells were injected subcutaneously in the flanks of the nude mice and tumor growth was assessed during biweekly treatment with DAPT or dimethyl sulfoxide (DMSO). After ~four weeks, at which point the maximum tumor size was ~1.5 cm^3^, the tumors were harvested and analyzed. The tumors derived from the colonospheres, parental and chemoresistant cells which were treated with DAPT were found to be significantly smaller than those treated with DMSO (control). However, the tumors derived from the colonospheres and chemoresistant cells exhibited significantly greater DAPT-induced growth inhibition compared with the parental cells. The 5FU-resistant and oxaliplatin-resistant cells showed 52 and 67% growth inhibition, respectively, while growth of the colonosphere cells was inhibited by 71% compared with the parental cells (21%; P<0.05; Fig. 4A).

**Figure 5:**
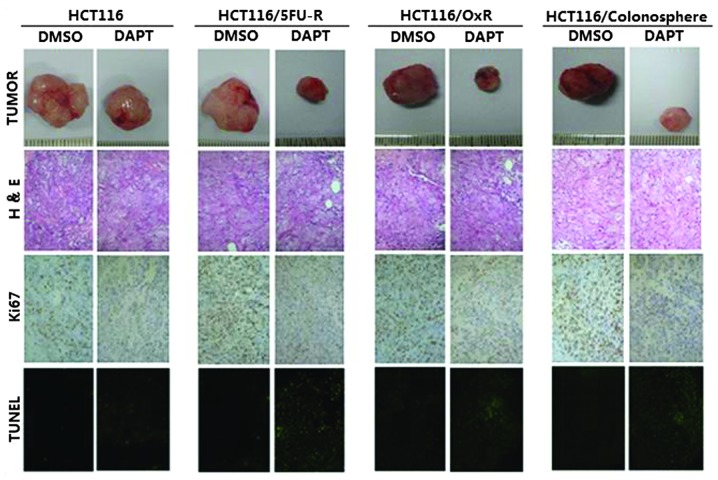
Effect of Notch pathway inhibition on *in vivo* tumor characteristics. Immunohistochemical analysis of tumors was performed and multiple tumor fields were analyzed per group. Representative images of all groups and treatments are presented (magnification, x100). H&E staining revealed similar subcutaneous tumor morphology among all groups of tumors. Ki67 staining showed decreased cell proliferation in the tumors treated with DAPT; however, no significant differences were observed in the cell proliferation between the HCT116-, colonosphere-, HCT116/5FU-R- and HCT116/Ox-R-derived tumor sections. TUNEL staining revealed significantly increased apoptosis in response to DAPT in colonosphere-, HCT116/5FU-R- and HCT116/Ox-R-derived tumors compared with tumors derived from HCT116 cells (P<0.05). 5-FU, 5-fluorouracil; R, resistant; Ox, oxaliplatin; DAPT, *N*-[*N*-(3,5-difluorophenacetyl)-l-alanyl]-*S*-phenylglycine *t*-butyl ester; DMSO, dimethyl sulfoxide; TUNEL, terminal deoxynucleotidyltransferase-mediated dUTP nick-end labeling; H&E, hematoxylin and eosin.

Analysis of the proliferation marker Ki67 using tumor section staining revealed that DAPT caused a decrease in the number of proliferating cells in all of the tumors compared with those treated with DMSO. Similar to tumor growth inhibition, the inhibition of Notch signaling had a greater effect on the tumors derived from the colonospheres and chemo-resistant cells than on the tumors derived from the parental cells; however, this difference was not found to be statistically significant. TUNEL staining was used to analyze apoptosis in the xenografts. Quantification of TUNEL staining showed that DAPT treatment caused significantly more apoptosis in the tumors derived from the colonospheres and chemoresistant cells compared with those derived from the HCT116 cells (P<0.05). Specifically, upon Notch signaling inhibition, HCT116 tumors showed a 2.1-fold increase in apoptotic nuclei compared with 5.4-, 5.9- and 5.7-fold increases in the HCT116/5FU-, HCT116/OxR- and colonosphere-derived tumors, respectively (all P<0.05; Fig. 4B). Representative images from the analyzed tumor sections and the subcutaneous tumors are shown in Fig. 5.

## Discussion

CRC is the second leading cause of cancer-associated mortality worldwide. Despite recent therapeutic regimens which have markedly increased survival in CRC, almost all CRC tumors become chemoresistant (24). Thus, it is necessary to understand the mechanisms of resistance in order to improve current treatment protocols in CRC.

The eradication of drug-resistant Co-CSCs is an important area of investigation (25). Numerous studies have used fluorescence-activated cell sorting (FACS) in order to identify and isolate CSCs. In the present study, FACS was not performed on CSCs as no reliable surface markers are available for identifying Co-CSCs. However, CSCs have the capacity to form colonies, also know as spheres, when cultured in the absence of serum. Therefore, colonosphere formation was used to investigate the characteristics of Co-CSCs (26). Chemoresistance is an important feature of Co-CSCs, thus the present study aimed to investigate the interrelation between Co-CSCs and chemoresistant cells. The present study focused on colonospheres and two chemoresistant cell lines and identified certain common features. Colonospheres and chemoresistant cells were found to be significantly enriched in the CSC markers CD133 and CD44 (27). This finding suggested that colonospheres may be abundant in Co-CSCs. Furthermore, the chemoresistance imparted in the two chemoresistant cell lines may be due to the acquisition of CSC phenotypes in these cell lines. Thus, in the present study, to further investigate the characteristics of Co-CSCs, colonospheres were cultured in serum-free media and chemoresistant cell lines were developed.

Colonospheres and chemoresistant cells were also found to be more quiescent *in vitro*, with decreased cell proliferation compared with the parental cells. However, a clonogenic assay revealed that colonospheres and chemoresistant cells had an increased capacity to form colonies and spheres in specialized serum-free media, characteristics which are consistent with the CSC phenotype (5). Furthermore, lysates obtained from cell line-derived colonospheres were observed to have increased resistance to 5FU and oxaliplatin as compared with the adherent parental cells. Oxaliplatin-resistant cells and 5FU-resistant cells also exhibited cross-resistance to 5FU and oxaliplatin, respectively. This suggested that colonospheres and chemoresistant cells activated general resistance pathways leading to multi-drug resistance. Colonospheres and chemoresistant cells were also found to be enriched in CSC markers and properties, consistent with the CSC phenotype. It is most likely that the process of developing colonospheres and chemoresistant cell lines involved increasing the expression of such CSC markers, rather than enriching the population of cells which already expressed these markers. Assessing this hypothesis may be difficult; however, previous studies have shown that the tumor microenvironment, including soluble factors and hypoxia, may affect colonosphere characteristics (28,29). However, little is known about the pathway involved in the expression of CSC markers in Co-CSCs and chemoresistant cells and further investigations into the mechanism of resistance and increased marker expression are required.

Therefore, the present study aimed to investigate the pathways involved in CSCs and chemoresistant cells and aimed to identify potential targets in the colonospheres and chemoresistant cells which would allow specific targeting of these cells with optimal agents. One such pathway was the Notch signaling pathway, which is involved in CRC progression and growth (30,31). The Notch1 gene copy number has been reported to be increased in colorectal adenocarcinomas and to be correlated with aggressive tumor behavior and poor prognosis (32–34). In the present study, constitutive Notch1 expression was observed to be higher in the colonospheres and chemoresistant cell lines compared with the parental cell lines, and most markedly increased in the colonospheres. Furthermore, the increase in Hes1 was associated with an increase in the levels of the targeted gene. In the present study, the Notch signaling pathway, particularly Notch1, was found to have a key role in colonospheres and chemoresistant cell lines, and it was hypothesized that the activation of Notch was involved in maintaining a phenotypic characteristic in the colonospheres and chemoresistant cell lines. However, the specific mechanism underlying the increase in the Notch1 expression in these cells has yet to be elucidated. The inhibition of Notch signaling *in vitro* was found to cause a decrease in cell growth, as determined by CCK-8 assay, and these effects were observed to be greater in the colonospheres and chemoresistant cell lines than in the parental cells. These findings showed that the inhibition of the Notch pathway using DAPT significantly depleted the number of colonosphere cells and chemoresistant cells, further validating the hypothesis that the activation of Notch is necessary for the maintenance of phenotypic characteristics in colonospheres and chemoresistant cell lines. *In vivo*, following DAPT treatment, the inhibition of the growth of colonosphere- and chemoresistant cell-derived tumors was found to be significantly higher than that of the parental cell-derived tumors. This finding suggested that the Notch signaling pathway may be important for Co-CSC maintenance and tumor resistance to standard chemotherapeutics. A previous study reported that colonic cancer cells may upregulate Notch1 as a protective mechanism in response to chemotherapy (35). Furthermore, in the present study, Notch inhibition was found to have a greater effect on the growth of colonosphere- and chemoresistant cell-derived tumors than parental cell-derived tumors *in vivo*, which was largely due to an increase in apoptosis. It is unlikely that colonospheres and chemoresistant cells acquired common molecular alterations to resist certain agents. However, the present study found that the colonospheres and chemoresistant cells acquired similar molecular and phenotypic alterations when cultured in serum-free media or when chronically exposed to chemotherapeutic agents. Of note, the colonospheres and chemoresistant cells also exhibited increased Notch1 and Hes1 expression, which led to these cells becoming more sensitive to the inhibition of the Notch pathway. Previous studies have suggested that targeting the Notch signaling pathway may be an effective method for targeting CSCs and chemoresistant cells (36,37). The findings of the present study suggest that inhibiting the Notch pathway using DAPT may be an effective strategy for targeting Co-CSCs and overcoming the chemoresistance of CRC cells in a clinical setting.
